# Comparative Transcriptomics Reveals Hypoxia-Modulated Transcriptional Responses to *Vibrio parahaemolyticus* Infection in *Takifugu obscurus*

**DOI:** 10.3390/ani16203174

**Published:** 2026-10-10

**Authors:** Jiachang Xu, Chen Long, Siyi Liu, Haoran Lin, Yong Zhang, Xianpei Huang, Danqi Lu

**Affiliations:** 1 School of Life Sciences and State Key Laboratory of Biocontrol, Southern Marine Science and Engineering Guangdong Laboratory (Zhuhai), Guangdong Provincial Key Laboratory for Aquatic Economic Animals and Guangdong Provincial Engineering Technology Research Center for Healthy Breeding of Important Economic Fish, Institute of Aquatic Economic Animals, Sun Yat-Sen University, Guangzhou 510275, China; xujch36@mail.sysu.edu.cn (J.X.); longch5@mail2.sysu.edu.cn (C.L.); liusy97@mail2.sysu.edu.cn (S.L.); lsslhr@mail.sysu.edu.cn (H.L.); lsszy@mail.sysu.edu.cn (Y.Z.); 2 Shanwei Marine Industry Institute, Shanwei Polytechnic, Shanwei 516600, China

**Keywords:** hypoxia, *Takifugu obscurus*, *Vibrio parahaemolyticus*, inflammation, pro-inflammatory cytokines

## Abstract

The disease triangle posits that outbreaks arise from host–pathogen–environment interactions. In aquaculture, hypoxia and *Vibrio* infection often co-occur, yet how dissolved oxygen influences antibacterial immunity remains unclear. We examined how hypoxia and *Vibrio parahaemolyticus* infection, individually and in combination, reshape the liver transcriptome of *Takifugu obscurus*. Shared responses to hypoxia and infection included *il15ra*, *smpd1*, *itga2*, *prkcb*, *map3k5*, *tp53inp1*, and *prdm1*, involved in immune activation, phagocytosis, lymphocyte development, inflammation, and apoptosis. Under infection, hypoxia was associated with altered expression of inflammatory regulators (*tcim*, *src*, *klf15*, *serpina3k*, *f2r*, *egr1*, *cebpb*) and modules of heat shock, phosphatases, circadian rhythm, and cell cycle/survival. A 2 × 2 factorial analysis identified 15 genes with significant oxygen × infection interactions, and the number of infection-responsive DEGs was markedly reduced under hypoxia (592 vs. 11), indicating that hypoxia reshapes rather than simply adds to the infection response. We also re-analyzed previously published metabolomic data from our earlier study as supporting evidence; this re-analysis revealed changes in prostaglandin A_2_, cuminaldehyde, and long-chain acylcarnitines, which may be associated with inflammatory regulation. In vitro, hypoxia modulated infection-induced cytokine transcription: at 3 h post-infection, hypoxia suppressed infection-induced *tnfa*, *il1b*, and *il6*; at 6 h, oxygen × infection interactions were detected for *tnfa* and *il1b*, whereas *il6* remained oxygen-insensitive. Collectively, this comparative transcriptomic analysis reveals hypoxia-modulated transcriptional responses to *V. parahaemolyticus* infection in *T. obscurus*, suggesting that hypoxia may perturb immune homeostasis and disease resistance in fish.

## Simple Summary

In aquaculture, low oxygen levels and bacterial infections often occur together, but how they interact to affect fish health is not well understood. We studied the liver of pufferfish (*Takifugu obscurus*) exposed to low oxygen and infection with *Vibrio parahaemolyticus*, a common bacterium in marine farming. We found that both low oxygen and infection changed many genes related to immunity and inflammation. When fish were infected, low oxygen altered how the liver responded to the bacteria, including regulating the expression of some inflammation-related genes. Our re-analysis also revealed changes in certain metabolites that may be linked to inflammation. These results suggest that low oxygen may disturb the immune balance of fish and make them more vulnerable to disease. Maintaining adequate oxygen in aquaculture systems may therefore help fish defend against infections. Our findings provide a basis for better disease management, but further work is needed to confirm the roles of the key genes identified here.

## 1. Introduction

The “disease triangle” emphasizes that disease occurrence depends on the combined effects of three factors: host, pathogen, and environment. Abnormal changes in any of these factors may disrupt the original balance, leading to disease outbreak and epidemic [1]. In the epidemiological analysis of aquatic diseases, ignoring the dynamic changes in complex aquatic environmental factors may make it difficult to reproduce the natural process of disease occurrence under laboratory conditions, because abiotic stressors (unsuitable dissolved oxygen, temperature, salinity and the like) are key drivers that trigger diseases. Most fish efficiently obtain oxygen through the countercurrent exchange system of their gills, and thus maintain high-frequency respiration via the continuous flow of water over the gills during locomotion, which means that fish often fail to acquire sufficient oxygen once their locomotor ability is impaired directly or indirectly by infection. *Flavobacterium columnare* (Bernardet and Grimont 1989) Bernardet et al. 1996 causes columnaris disease in fish, usually resulting in skin lesions, fin erosion and gill necrosis [2]; nervous necrosis virus leads to histopathological lesions characterized by vacuolization and necrosis in the central nervous system of fish, and also impairs locomotion, swim bladder control and vision [3]; *Cryptocaryon irritans* Brown, 1951 invades the skin, fins, gills and eyes of marine fish, causing lethargy and suffocation [4]. Taken together, the damage to the functions of organs such as gills, fins, brain and swim bladder caused by pathogens means that fish may be exposed to hypoxia even in water bodies with high dissolved oxygen levels. Furthermore, during the invasion of pathogens from the environment into the host, the physiological conditions of the “immune microenvironment” where pathogen–immune cell interactions occur are largely altered. Pathogens carry out aerobic respiration [5], and the recruited immune cells after infection themselves consume oxygen to produce reactive oxygen species (ROS) and accelerate cellular metabolism to promote the synthesis of inflammatory mediators. These factors collectively render the immune microenvironment hypoxia. Accordingly, accounting for oxygen availability is imperative during disease outbreaks in aquaculture systems.

Given the co-occurrence of hypoxia and pathogen infection, it is not surprising that organisms need to adapt their immune responses under hypoxic conditions. Nuclear factor-κB (NF-κB) regulates the expression of many pro-inflammatory cytokines involved in immune and inflammatory responses, such as tumor necrosis factor (TNF), interleukin-1 (IL-1) and IL-6 [6]. The canonical NF-κB pathway is rapidly triggered by pro-inflammatory cytokines, and by pathogen or damage-associated molecular patterns [7], and then activates transforming growth factor-beta1 (TGF-β1)–activated kinase 1 (TAK1), which contributes to the activation of a trimeric IκB kinase (IKKα, IKKβ and IKKγ) complex by the phosphorylation of IKKβ. The activated IKK complex then phosphorylates IκBα or other IκB family members, which leads to the ubiquitylation and proteasomal degradation of IκB, resulting in the release and nuclear translocation of the canonical NF-κB family members, predominantly the NF-κB1 p50-RelA and NF-κB1 p50-c-Rel dimers [8]. As a rapid response factor [9], NF-κB is also a critical transcriptional activator of hypoxia-inducible factor 1 (HIF-1), and this links innate immunity to the hypoxic response [10,11,12]. In addition to the NF-κB pathway, recent studies in murine and zebrafish models have revealed that oxygen availability directly shapes innate immunity through hydroxylation modifications of key signaling molecules. Cai et al. demonstrated that factor inhibiting HIF (FIH) negatively regulates antiviral responses by hydroxylating IκB kinase ε (IKKε), thereby disrupting type I interferon production [13]. Conversely, Liu et al. showed that EGLN1 (egg-laying-defective nine homolog 1), an oxygen-sensing prolyl hydroxylase, positively potentiates antiviral immunity by hydroxylating interferon regulatory factor 3 (IRF3), promoting its activation and subsequent interferon induction [14]. These findings establish a conserved role for oxygen, via hydroxylase-mediated modifications, in regulating innate immune signaling across vertebrates.

While it is increasingly recognized that oxygen ultimately shapes antiviral immunity by modulating the expression of host defense genes, how hypoxia affects these defense genes and their upstream regulatory nodes—particularly in the context of antibacterial immunity—remains poorly understood in fish. *Vibrio* species are widely distributed in various aquatic habitats, and high abundances of *Vibrio* can be detected in the tissues or organs of many marine organisms, including seaweeds, corals, fish, shrimp, bivalves, sponges, abalones, squids and zooplankton [15]. Many *Vibrio* species have been reported to be highly pathogenic to marine organisms, and the resulting vibriosis has caused substantial economic losses to aquaculture [16]. Among them, *Vibrio parahaemolyticus* (Fujino et al. 1951) Sakazaki et al. 1963 is one of the most important bacterial pathogens in marine aquaculture and a well-established model for studying bacterial virulence and host antibacterial immunity [16]. Pufferfish serve as an important model for comparative genomic research in vertebrates [17], and *Takifugu obscurus* (Abe, 1949) is sensitive to both *Vibrio* infection and hypoxia [18,19]. In this study, comparative transcriptomic analyses were used to identify candidate genes potentially associated with disease resistance and regulated by oxygen availability, many of which participate in the modulation of inflammatory responses. In vitro infection assays revealed that the transcriptional responses of defense genes encoding pro-inflammatory cytokines *tnfa* and *il1b* against *Vibrio* challenge are modulated by oxygen levels. This suggests that severe hypoxia may be associated with disruption of the canonical regulatory control of these molecules and may impair immune homeostasis under both healthy and pathogenic conditions.

## 2. Materials and Methods

### 2.1. Animals, Treatments of Stressors and Sample Collection

The relevant information is as described previously [20]. A total of 160 juvenile pufferfish (*Takifugu obscurus*) (approximately 8.0 cm in length, 12 months old) were purchased from Guangzhou Jinyang Aquaculture Co., Ltd. (Guangzhou, China). The fish were acclimated under laboratory conditions for 2 weeks and fed commercial feed twice daily before the experiment. They were randomly divided into four groups, with four tanks per group and ten fish per tank (30 L water per tank). Each group was exposed to normoxia (7.60 ± 0.20 mg/L) or hypoxia (2.50 ± 0.20 mg/L) for 24 h at 25.0 ± 0.5 °C, and dissolved oxygen concentrations were continuously controlled by bubbling nitrogen or oxygen into the tanks. At 12 h post-exposure, fish were intraperitoneally injected with PBS or *V. parahaemolyticus* RIMD 2210633 (5 × 10^7^ colony-forming units, CFU). At 24 h, one fish was randomly selected from each tank (*n* = 4 per group), and liver tissue pieces of approximately 0.5 cm^3^ were sampled, immediately snap-frozen in liquid nitrogen, and stored at −80 °C until RNA extraction for transcriptomic analysis.

### 2.2. Cells, Strains and Culture Conditions

The Fugu cell line (CRL-2641) was purchased from ATCC (Manassas, VA, USA). The cells were cultured in a customized medium consisting of 67.5% DMEM (Invitrogen, Carlsbad, CA, USA), 25% L-15 (Hyclone, Logan, UT, USA) and 7.5% F12 (Invitrogen). The medium was supplemented with 0.5 g/L sodium bicarbonate (Invitrogen), 15 mM HEPES (Invitrogen) and 0.5 mM GlutaMAX™ (Invitrogen). Prior to use, 0.05 mM 2-mercaptoethanol (Invitrogen) and 10% fetal bovine serum (Invitrogen) were added. The wild-type strain and the type III secretion system 1 (T3SS1)-inactivated mutant (Δ*exsA*) of *V. parahaemolyticus* were preserved in our laboratory. All of the *V. parahaemolyticus* strains were cultured in Miller’s Luria–Bertani broth supplemented with 3% (*w*/*v*) NaCl at 30 °C.

### 2.3. In Vitro Infection Methods

Infection assays were performed as previously described [21]. Briefly, Fugu cells were seeded into 6-well plates at a density of 4 × 10^5^ cells per well one day prior to infection and incubated overnight to allow cell attachment; three independent biological replicates were prepared for each experimental condition. Meanwhile, *V. parahaemolyticus* strains were revived. On the following morning, bacterial strains were further activated. Cell monolayers were washed twice with basal medium, followed by the addition of *Vibrio*-containing infection medium at a multiplicity of infection (MOI) of 10 to each well. Multi-well plates were centrifuged at 183× *g* for 5 min at room temperature to synchronize bacterial infection. Subsequently, plates were gently transferred to incubators maintained at 22 °C under 21% or 1% oxygen tension, respectively. At designated time-points post-infection, plates were harvested, and samples were processed according to downstream experimental requirements.

### 2.4. Quantitative Real-Time Polymerase Chain Reaction Analysis

Total RNA of Fugu cells was extracted using the FastPure Cell/Tissue Total RNA Isolation Kit V2 (Vazyme, Nanjing, China). Reverse transcription was performed using HiScript IV All-in-One Ultra RT SuperMix for qPCR (Vazyme). qPCR was carried out using SupRealQ Purple Universal SYBR qPCR Master Mix (U+) (Vazyme). All qPCR measurements were derived from three independent biological experiments. Each independent experiment included three biological replicates, and every biological replicate was assayed in three technical replicate wells. Primer specificity was preliminarily verified by electrophoresis and melting curve analysis (Table 1). No-template controls (NTCs) were included in each qPCR run; no amplification product was detected in NTCs by melting curve analysis, indicating specific amplification. All primers were designed to span exon–exon junctions, and RNA samples were treated with DNase I; therefore, no-reverse-transcriptase (NRT) controls were not included. Since the mRNA expression levels of the three pro-inflammatory cytokines were extremely low under normal conditions, the coding sequences of the three pro-inflammatory cytokines were cloned into plasmids for the primer amplification efficiency test. The plasmids were used as templates for 5-fold serial dilution.

*rpl13* was selected as the reference gene for qPCR analyses across all experimental groups, as it is relatively stable under nutrient limitation and in different tissues [22]. As validated in Figure S1, the stability of *rpl13* was assessed by comparing its CT values across all experimental groups; *rpl13* remained stable under hypoxia, *V. parahaemolyticus* infection, and combined hypoxia and infection stressors (equal amounts of cDNA were input). Analyses were performed on the QuantStudio™ 7 Flex (Applied Biosystems, Carlsbad, CA, USA) according to the manufacturer’s instructions.

### 2.5. RNA Isolation, Library Preparation and Sequencing

Four fish from each treatment group were used as biological replicates for transcriptomic analysis. The livers were mechanically homogenized and total RNA was extracted using the RNAprep pure Tissue Kit according to the manufacturer’s instructions and treated with DNase I (TIANGEN Biotech, Beijing, China). RNA integrity and total amount were accurately detected by an Agilent 2100 Bioanalyzer (Agilent Technologies, Santa Clara, CA, USA). According to the instructions of the NEBNext Ultra RNA Library Prep Kit for Illumina (New England Biolabs, Ipswich, MA, USA), mRNA was purified using poly-T oligo-attached magnetic beads. Fragmentation was used for cDNA synthesis, and the cDNA was purified to preferentially select fragments 370 to 420 bp in length using the AMPure XP system (Beckman Coulter, Indianapolis, IN, USA). Then PCR was performed with universal PCR primers and index (X) Primer. Finally, quantified libraries were subjected to 150 bp paired-end sequencing on the Illumina NovaSeq platform (Illumina, San Diego, CA, USA).

### 2.6. Data Processing and Analysis

Clean data were obtained by removing reads containing adapters, reads containing poly-N sequences and reads of poor quality from the raw data. Given the absence of a publicly available reference genome for *T. obscurus* on NCBI, we adopted the genome of *Takifugu rubripes* (Temminck & Schlegel, 1850), a closely related species with high genomic similarity, as the reference for read-mapping. Specifically, reads were mapped to the *T. rubripes* reference assembly GCF_901000725.2 (fTakRub1.2). Gene annotation was obtained from NCBI *T. rubripes* Annotation Release 103. Alignment statistics shown in Supplementary Table S1 revealed that the average total mapping rate of the 16 transcriptomic samples was 85.10%, which represented a good alignment performance for cross-species mapping. The paired-end clean reads were aligned to the genome of *T. rubripes* using open-source HISAT2 (v2.0.5) [23]. Gene-level raw read counts were generated using featureCounts (v1.5.0-p3) [24]. Differential expression analysis was performed using the raw count matrix as input with DESeq2 (v1.20.0) [25]. We first performed pairwise group-to-group comparisons to describe condition-specific transcriptional changes. Pairwise contrasts included hypoxic control versus normoxic control (HC vs. NC), normoxic infection versus normoxic control (NI vs. NC), hypoxic infection versus normoxic control (HI vs. NC), and hypoxic infection versus normoxic infection (HI vs. NI). To formally test whether the infection response depends on oxygen availability, we additionally fitted a 2 × 2 factorial DESeq2 model with the design ~ hypoxia * infection (equivalent to ~ oxygen + infection + oxygen:infection). The interaction term corresponded to (HI − HC) − (NI − NC), and genes with adjusted *p*-value (padj) < 0.05 for this term were considered to have a significant oxygen × infection interaction. The complete interaction results are provided in Supplementary Table S6. DEG lists for the four simple effects are provided in Supplementary Tables S7–S10: Table S7 (NI vs. NC, normoxic infection effect), Table S8 (HI vs. HC, hypoxia infection effect), Table S9 (HC vs. NC, control hypoxia effect), and Table S10 (HI vs. NI, infected hypoxia effect). Additionally, fragments per kilobase of transcript per million mapped reads (FPKM) values were calculated to facilitate data visualization. Genes with |log_2_(fold change)| ≥ 1 and adjusted *p*-value (padj) < 0.05 were considered significantly differentially expressed. Visualizations were performed using the OmicShare tools (https://www.omicshare.com/tools (accessed on 30 June 2026)).

Metabolomic and transcriptomic samples were obtained from independent experiments. The metabolomic data were derived from our previously published study [20] and are re-analyzed here as supporting evidence. In brief, liver homogenate (100 mg) was extracted with 500 μL prechilled 80% methanol/0.1% formic acid, incubated on ice, centrifuged, diluted to 53% methanol, and centrifuged again. LC–MS was performed on a Vanquish UHPLC system coupled to an Orbitrap Q Exactive HF-X mass spectrometer with a Hypesil Gold C18 column (100 × 2.1 mm, 1.9 μm) using a 17 min gradient at 0.2 mL/min in positive and negative modes. Pooled QC samples were injected periodically to monitor instrument stability. Peak intensities were normalized by total peak area. Metabolites were annotated against mzCloud, mzVault, and MassList, and further annotated using KEGG, HMDB, and LIPID MAPS. Identification confidence was MSI Level 2 (putative annotation by MS/MS spectral matching). PCA and PLS-DA were performed using metaX (v1.4.16), with permutation test for PLS-DA validation. Candidate differential metabolites were screened by VIP > 1.0, fold change > 1.20 or <0.83, and raw *p* < 0.05 (Student’s *t*-test). No FDR correction was applied; these metabolites are therefore considered exploratory candidates.

The mRNA levels of target genes were normalized to the internal reference *rpl13* and expressed as ΔCT values (CT_target − CT_*rpl13*) for all statistical analyses. To facilitate data visualization, relative fold changes (2^−ΔΔCT^) were calculated and presented in the figures [26], with the normoxic control group serving as the calibrator.

All qPCR data were obtained from three independent experiments, each consisting of three biological replicates, and each biological replicate was assayed with three technical replicates. For each gene and time point, the ΔCT values from the three biological replicates were averaged to obtain a single representative value per independent experiment, yielding three independent data points per experimental group (*n* = 3 independent experiments).

Statistical significance was assessed using aligned rank transformed two-way analysis of variance (ART ANOVA) with experiment as a random factor, followed by Tukey’s honest significant difference (HSD) post hoc tests for significant interactions. The main effects of oxygen tension (normoxia vs. hypoxia), bacterial infection (control vs. *V. parahaemolyticus*), and their interaction were evaluated. A *p*-value < 0.05 was considered statistically significant. All analyses were performed using the ARTool package (v0.11.2) in R (v4.2.0) [27,28].

## 3. Results

### 3.1. Hypoxia and V. parahaemolyticus Infection Trigger Overlapping Transcriptomic Signatures in Liver

A model of *T*. *obscurus* under hypoxia and infection with *V. parahaemolyticus* was established, and four groups were defined as the normoxic control (NC), hypoxic control (HC), normoxic infection (NI) and hypoxic infection (HI) groups. These groups of *T*. *obscurus* were exposed to normoxia or hypoxia for 24 h, and each individual was injected with PBS or *V. parahaemolyticus* at 12 h. As a high-oxygen-demanding organ that serves both metabolic and immune roles, the liver displayed obvious inflammatory manifestations relative to the spleen and head-kidney under the stressors [20]. Since peak mortality occurred at 36 h post-injection, liver samples were collected at 24 h to perform omics analyses before severe pathological deterioration (Figure 1a). Full lists of DEGs for the three pairwise comparisons (HC: NC, NI: NC, HI: NC) are provided in Supplementary Tables S2–S4, respectively. The Venn diagram of differentially expressed genes (DEGs) of the three compared groups (HC: NC, NI: NC, HI: NC) indicated that 49 DEGs were shared between the HC:NC and NI:NC contrasts; of these, 30 were also detected in the HI:NC contrast (Figure 1b). Among the 30 shared DEGs, a series of genes were identified as being involved in immune cell activation, phagocytosis, B-cell and T-cell development and differentiation, inflammatory response, and apoptosis, including interleukin-15 receptor subunit alpha (*il15ra*), sphingomyelin phosphodiesterase (*smpd1*), integrin alpha-2 (*itga2*), protein kinase C beta type (*prkcb*), mitogen-activated protein kinase kinase kinase 5 (*map3k5*), tumor protein p53-inducible nuclear protein 1 (*tp53inp1*), and PR domain zinc finger protein 1 (*prdm1*) (Figure 1c). Overall, the identification of DEGs indicated that hypoxia and/or *V. parahaemolyticus* infection induced overlapping immunostimulatory characteristics.

### 3.2. Transcriptomic Differences Between Normoxic and Hypoxic V. parahaemolyticus-Challenged T. obscurus

Although hypoxia and *V. parahaemolyticus* infection individually and jointly induced overlapping immunostimulatory signatures, the transcriptional changes specifically associated with hypoxia during infection remained to be defined. We therefore compared the significantly differentially expressed genes between the NI and HI groups. The full list of DEGs is provided in Supplementary Table S5. Importantly, seven molecules involved in regulating the expression of inflammatory cytokines were identified: transcriptional and immune response regulator (*tcim*), proto-oncogene tyrosine-protein kinase Src (*src*), Krueppel-like factor 15 (*klf15*), serine protease inhibitor A3K-like (*serpina3k*), coagulation factor II thrombin receptor (*f2r*), early growth response 1 (*egr1*), and CCAAT/enhancer-binding protein beta (*cebpb*). In addition to the key transcription factor AP-1 (*jun*) (Figure 2a), we further manually grouped the DEGs into four literature-based functional categories according to the known functions of their mammalian homologs (UniProt annotations and published reports), rather than from formal enrichment analysis. These categories were: the heat shock signaling module (including heat shock 70 kDa protein 1 (*hspa1*), DnaJ homolog subfamily B member 1 (*dnajb1*), heat shock protein family E (Hsp10) member 1 (*hspe1*), DnaJ homolog subfamily C member 1 (*dnajc1*), and heat shock protein nuclear import factor Hikeshi (*hikeshi*)), the phosphatases module (including dual specificity protein phosphatase 3 (*dusp3*), dual specificity protein phosphatase 7 (*dusp7*), and dual specificity protein phosphatase 5 (*dusp5*)), the circadian rhythm module (including class E basic helix-loop-helix protein 40 (*bhlhe40*), circadian-associated transcriptional repressor (*ciart*), and nuclear receptor subfamily 1 group D member 2 (*nr1d2*)), and the cell cycle, proliferation and survival module (including Krueppel-like factor 10 (*klf10*), breast cancer anti-estrogen resistance protein 3 (*bcar3*), cyclin-dependent kinase inhibitor 1B (*cdkn1b*), ornithine decarboxylase 1 (*odc1*), cyclin-G2 (*ccng2*), fructose-2,6-bisphosphatase TIGAR (*tigar*), tripartite motif-containing protein 16 (*trim16*), serine/threonine-protein kinase (*pim3*), insulin receptor substrate 2 (*irs2*) and its transcript variant X2 (*irs2-x2*)) (Figure 2b). In summary, we identified four functional modules (heat shock, phosphatases, circadian rhythm, and cell cycle/survival) and a set of inflammatory regulators (including *tcim*, *src*, *klf15*, *serpina3k*, *f2r*, *egr1*, and *cebpb*) that are differentially expressed under hypoxic conditions.

### 3.3. Differentially Expressed Metabolites Under Hypoxia or V. parahaemolyticus Infection Are Associated with Inflammatory Regulation

Importantly, a panel of inflammation-associated differential metabolites, which have already been reported in our previously published metabolomics analysis [20], were re-analyzed in the current study. Without supporting transcriptomic data in the earlier work, the upstream regulatory mechanisms and functional implications of these metabolites remained unclear. Taking the inflammation-related transcriptomic signatures identified in this study as a starting point, we revisited these metabolites and systematically categorized them into three functional groups. PGA_2_ (prostaglandin A_2_) and CA (cuminaldehyde) might contribute to anti-inflammatory effects (Figure 3a). Sphingomyelin and sphingosine are related to sphingolipid metabolism, and they are linked through the enzymatic activity of SMPD1, which hydrolyzes sphingomyelin to generate ceramide, the direct precursor of sphingosine (Figure 3b). The dynamics of long-chain acylcarnitines (LCACs) may be associated with fatty-acid oxidation and anti-inflammatory signaling (Figure 3c). Collectively, these metabolic alterations, together with the transcriptomic changes described above, preliminarily suggest that inflammatory signaling pathways may be modulated in response to *V. parahaemolyticus* infection and hypoxia.

### 3.4. Hypoxia Reshapes the Transcriptional Response to V. parahaemolyticus Infection: A 2 × 2 Factorial Analysis

To formally test whether the infection response depends on oxygen availability, we performed a 2 × 2 factorial DESeq2 analysis with the model ~ hypoxia * infection. The interaction term, defined as (HI − HC) − (NI − NC), identified 15 genes with significant non-additive responses (padj < 0.05; Supplementary Table S6), including *gas2l3*, *pdlim7*, *trmt2b*, *klf1*, *gata2*, *fgfr4*, *septin3*, *tspan8*, *klf2*, *itga2*, *slc25a25*, *zeb1b*, *lrrc40*, *sec14l1*, and *mllt3*. Notably, the number of infection-responsive DEGs was markedly reduced under hypoxia (592 under normoxia vs. 11 under hypoxia; Supplementary Tables S7 and S8), while hypoxia itself induced fewer DEGs in infected fish than in uninfected fish (56 for HI vs. NI vs. 429 for HC vs. NC; Supplementary Tables S9 and S10). These results indicate that hypoxia does not simply add to the infection response, but rather reshapes the transcriptional program of infected fish, potentially attenuating the magnitude of the antibacterial inflammatory response. Several of the interaction genes, including *tspan8*, *zeb1b*, and *sec14l1*, have mammalian homologs implicated in inflammatory regulation [29,30,31], suggesting that oxygen availability may remodel the transcriptional responses to *Vibrio* infection.

### 3.5. Reduced Oxygen Partial Pressure Modulates the mRNA Expression of Pro-Inflammatory Cytokines Induced by V. parahaemolyticus in Fugu Cells

A robust inflammatory response with elevated pro-inflammatory cytokine production constitutes a critical line of host defense against bacterial infection. We performed in vitro hypoxic infection assays to characterize how oxygen availability shapes the transcriptional induction of core pro-inflammatory mediators *tnfa*, *il1b* and *il6*. These three cytokines serve as key inflammatory mediators during infection and are widely used to indicate the progression of infectious diseases [32,33].

The T3SS1 of *Vibrio* rapidly kills eukaryotic cells in vitro [34]. We infected Fugu cells with *V. parahaemolyticus* with inactivated T3SS1, which extended the analytical time window to at least 6 h post-infection (hpi) (Figure S2). Normoxic and hypoxic conditions were set as 21% O_2_ (standard for routine cell culture) and 1% O_2_ (hypoxia), respectively, and we analyzed the expression of pro-inflammatory cytokines in this model (Figure 4a).

Data were analyzed using aligned rank transformed two-way ANOVA with experiment as a random factor, followed by Tukey’s HSD post hoc tests for significant interactions. The *p*-values for the main effects of oxygen (O), infection (I), and their interaction (O × I), as well as the specific pairwise comparison between NI and HI (where significant), are shown in Figure 4b,c.

At 3 hpi, *V. parahaemolyticus* challenge significantly elevated all three cytokines (main effect of infection, I: all *p* < 0.001). Significant oxygen × infection interactions were detected for all three genes (*tnfa*: *p* = 0.0042; *il1b*: *p* < 0.0001; *il6*: *p* = 0.0015). Under infectious conditions, hypoxia significantly dampened the infection-triggered induction of *tnfa* (NI vs. HI: *p* = 0.0490), *il1b* (NI vs. HI: *p* = 0.0020), and *il6* (NI vs. HI: *p* = 0.0144), indicating a broad early suppressive effect of hypoxia on the inflammatory response (Figure 4b).

At 6 hpi, infection continued to upregulate all three genes (main effect of infection, I: *tnfa* and *il1b*: both *p* < 0.001; *il6*: *p* = 0.0018). Significant oxygen × infection interactions were detected for *tnfa* (*p* = 0.0070) and *il1b* (*p* = 0.0092), but not for *il6* (*p* = 1.0000), suggesting potential oxygen-dependent effects on *tnfa* and *il1b* at this later time point. However, the direct NI vs. HI comparisons were not statistically significant for *tnfa* (*p* = 0.0784) or *il1b* (*p* = 0.1824). Therefore, these results should be interpreted cautiously and do not provide evidence for a significant direct effect of hypoxia on *tnfa* or *il1b* at 6 hpi. For *il6*, no significant oxygen × infection interaction (*p* = 1.0000) or oxygen main effect (*p* = 0.4738) was detected, indicating that *il6* was not significantly affected by oxygen at 6 hpi (Figure 4c).

Collectively, these in vitro results suggest that oxygen tension may modulate *V. parahaemolyticus*-induced *tnfa* and *il1b* transcription, with a significant suppressive effect at 3 hpi but no significant direct effect at 6 hpi; *il6* induction was not significantly affected by hypoxia at later times. These cell-based observations are consistent with our transcriptomic and metabolomic findings, and are consistent with the possibility that hypoxia may remodel host transcriptional responses to *Vibrio* infection.

## 4. Discussion

### 4.1. Molecular Convergence of Hypoxia and Infection Responses: Differential Gene Expression and Functional Interpretation of Molecular Responses

Under hypoxia or infection, a series of genes were significantly differentially expressed, including *il15ra*, *smpd1*, *itga2*, *prkcb*, *map3k5*, *tp53inp1*, and *prdm1*. Notably, the IL-15 signaling pathway serves as a core driver of NK and CD8^+^ T-cell immune responses, governing the critical processes of survival, proliferation, and activation [35]. Furthermore, SMPD1 is critical for antibacterial immunity by driving efficient phagosome–lysosome fusion in macrophages, a function achieved via the hydrolysis of sphingomyelin to ceramide and phosphorylcholine [36]. Similarly, during *Listeria monocytogenes* (Murray et al. 1926) Pirie 1940 infection, the α_2_ integrin subunit encoded by *Itga2* in mast cells plays an important immune protective role by mediating inflammatory responses [37]. By contrast, PRKCB exacerbates *Mycobacterium avium* Chester 1901 infection and promotes intracellular bacterial survival by inhibiting the fusion of lysosomes with mycobacterial phagosomes [38]. Moreover, MAP3K5 mediates stress-induced apoptotic signaling and plays a critical role in antibacterial host defense [39]. In addition, TP53INP1 promotes autophagy-dependent cell death [40]. Finally, B-lymphocyte-induced maturation protein 1 (Blimp-1), encoded by the *PRDM1* gene, broadly regulates the functions of various immune cells, including T cells and dendritic cells, and plays a central role in maintaining immune homeostasis and tissue tolerance [41].

Collectively, these expression changes highlight a functional convergence between hypoxic and infectious contexts. With regard to the similarity in molecular responses exhibited by fish under hypoxia and *Vibrio* infection, one possible explanation is that hypoxia and infection are not independent events but are closely intertwined physiologically and ecologically. On the one hand, infection itself can induce local tissue hypoxia: the inflammatory response typically requires the recruitment of large numbers of myeloid cells, and the accompanying high metabolic burden and the enormous energy demand of cell migration dramatically increase local oxygen consumption [42,43,44]; meanwhile, invading pathogens (e.g., *Shigella flexneri* Castellani and Chalmers 1919) can also consume substantial oxygen at the infection site through aerobic respiration, directly causing “infectious hypoxia” [5]. Consequently, during the course of infection, host cells are in effect exposed to a hypoxic microenvironment, making the activation of hypoxia-response pathways an intrinsic component of the infection response. On the other hand, from an evolutionary perspective, given that hypoxia and pathogen invasion often co-occur in nature, fish may have, over long-term adaptation, deeply integrated immune defense responses into the hypoxia-response network, thereby exhibiting shared regulatory features at the molecular level.

### 4.2. Hypoxia-Associated Alterations in Immune Molecule and Metabolite Homeostasis: Potential Links to Disease Outbreaks in Fish

Specifically, seven molecules (*tcim*, *src*, *klf15*, *serpina3k*, *f2r*, *egr1*, and *cebpb*) known or predicted to regulate inflammatory cytokine expression were differentially expressed under hypoxia during infection. Among them, TC1 (encoded by *TCIM*) is upregulated by IL-1β, TNF-α, LPS, and phorbol ester, and it enhances NF-κB nuclear translocation and DNA-binding activity [45]. In addition, Src kinase activates IKKα/IKKβ and IκBα through tyrosine phosphorylation, thereby acting upstream to regulate and initiate both the canonical and atypical (IKK-independent) NF-κB pathways [46]. Furthermore, KLF15 can alter the acetylation status and activity of the pro-inflammatory factor NF-κB [47]. In contrast, SERPINA3K inhibits inflammation induced by retinal ischemia/hypoxia by attenuating intracellular oxidative stress [48]. Similarly, PAR-1 (encoded by *F2r*) protects the host from *Helicobacter pylori* (Marshall et al. 1985) Goodwin et al. 1989-induced gastritis by suppressing the production of pro-inflammatory cytokines [49]. On the other hand, LPS-induced EGR1 gene expression is necessary for maximal induction of TNF-α in monocytes [50]. Finally, c/EBPβ (encoded by *Cebpb*) drives robust expression of pro-inflammatory genes in microglia [51].

Beyond these direct inflammatory regulators, hypoxia was also associated with altered expression of a broader set of cellular stress and immune modulatory pathways that collectively maintain immune homeostasis. Heat shock proteins maintain cellular homeostasis under stress conditions by promoting correct protein folding, degrading damaged proteins, and clearing protein aggregates [52]; notably, the expression of the HSP70 gene is also induced by hypoxia [53]. Likewise, MAPK is a central signaling node that determines cell fate—proliferation, differentiation, survival, apoptosis, chemotaxis, and inflammatory mediator production—while the strength and persistence of this signal are dynamically controlled by dual-specificity phosphatases, which act by dephosphorylating phosphothreonine/phosphotyrosine residues [54]. Moreover, the immune system is under circadian regulation, with white blood cells, for instance, peaking in the circulation during the rest phase [55]. Consistently, a human blood transcriptome study demonstrates that hypoxic exposure not only modifies clock gene expression but also makes the daily oscillations of immune-related genes and cell abundance altitude-dependent [56], implying that hypoxia may modulate immune responses through circadian disruption. In parallel, similar to the induction of heat shock protein pathways, genes related to cell cycle, proliferation, and survival were differentially expressed under hypoxia, which may contribute to cell survival. From the pathogen’s perspective, *Vibrio* may employ activation of survival pathways and inhibition of cell death pathways as one of the strategies to suppress host immune responses [57].

Additionally, based on the metabolomic data re-analyzed from our previously published study [20], LCACs may stimulate the mRNA expression and secretion of *tnfa*, *il1b*, and *il6* [58], whereas PGA_2_, a prostaglandin containing a cyclopentenone moiety, may inhibit TNF-α-induced IKK and NF-κB activities [59,60], and CA may exert anti-inflammatory effects by blocking the NF-κB signaling pathway [61].

Collectively, the 2 × 2 factorial analysis revealed a significant statistical interaction between hypoxia and infection, suggesting that the transcriptional response to *V. parahaemolyticus* may depend on oxygen availability, particularly with respect to inflammatory responses. Indeed, the induction of interference with pro-inflammatory transcriptional responses is a core strategy by which pathogens evade immunity [62]. For example, the *V. parahaemolyticus* effector protein VopS binds to the host cytoplasmic RELA-p65/NF-κB and inhibits NF-κB transcriptional activity [63]. Similarly, VopZ selectively blocks the formation of the host TAK1 activation complex, thereby inhibiting the activation of MAPK and NF-κB signaling pathways [64]. Correspondingly, the mRNA expression of *tnfa* and *il1b*, which are downstream of the NF-κB pathway, differed under hypoxia, consistent with a potential role of oxygen in the anti-*Vibrio* immune inflammatory response.

Several limitations should be acknowledged. First, reads were mapped to the *T*. *rubripes* genome due to the lack of a *T*. *obscurus*-specific reference, which may introduce cross-species mapping bias and affect differential-expression estimates. Second, the in vitro experiments using the T3SS1-inactivated strain provide supportive mechanistic evidence rather than direct validation of the in vivo model, because T3SS1 disruption may alter host–pathogen interactions and the wild-type strain’s severe cytotoxicity may mask hypoxia-related inflammatory regulation.

Taken together, in this study, we identified a set of candidate oxygen-associated molecules potentially associated with antibacterial defense and disease resistance in fish. These hypoxia-regulated molecules are known or predicted to participate in antibacterial immunity. Therefore, excessive hypoxia may be associated with disrupted immune homeostasis in both normal and diseased states, and with altered expression of these molecules.

## 5. Conclusions

This study shows that both hypoxia and *V. parahaemolyticus* infection were associated with altered expression of inflammatory regulators and immune-related functional modules in *T. obscurus* liver. Shared and infection-associated responses converged on immune cell activation, phagocytosis, lymphocyte development, inflammation, and apoptosis. Under infection, hypoxia was further associated with immunologically relevant stress and regulatory modules, including heat shock, phosphatase, circadian rhythm, and cell cycle/survival pathways, which are linked to stress–immune crosstalk and immune cell function. A 2 × 2 factorial analysis identified genes with significant oxygen × infection interactions, indicating that hypoxia reshapes rather than simply adds to the infection response. We also re-analyzed previously published metabolomic data from our earlier study as supporting evidence; this re-analysis revealed changes in prostaglandin A_2_, cuminaldehyde, and long-chain acylcarnitines, which may be associated with inflammatory regulation. These transcriptomic changes, together with the re-analyzed metabolomic findings, suggest that oxygen availability may influence antibacterial inflammatory responses and prompted functional examination of infection-induced pro-inflammatory cytokines. In Fugu cells, hypoxia modulated *V. parahaemolyticus*-induced *tnfa*, *il1b*, and *il6* transcription. Early hypoxia suppressed infection-induced cytokine induction, whereas at later infection oxygen × infection interactions were detected for *tnfa* and *il1b*, while *il6* remained largely oxygen-insensitive. Collectively, this comparative transcriptomic analysis reveals hypoxia-modulated transcriptional responses to *V. parahaemolyticus* infection in *T. obscurus*. These findings suggest that dissolved oxygen, together with bacterial infection, may influence immune homeostasis and potentially affect disease resistance in fish, providing a framework for understanding environmental oxygen effects on aquaculture disease outbreaks. Functional validation of key nodes is needed to establish causality.

## Figures and Tables

**Figure 1:**
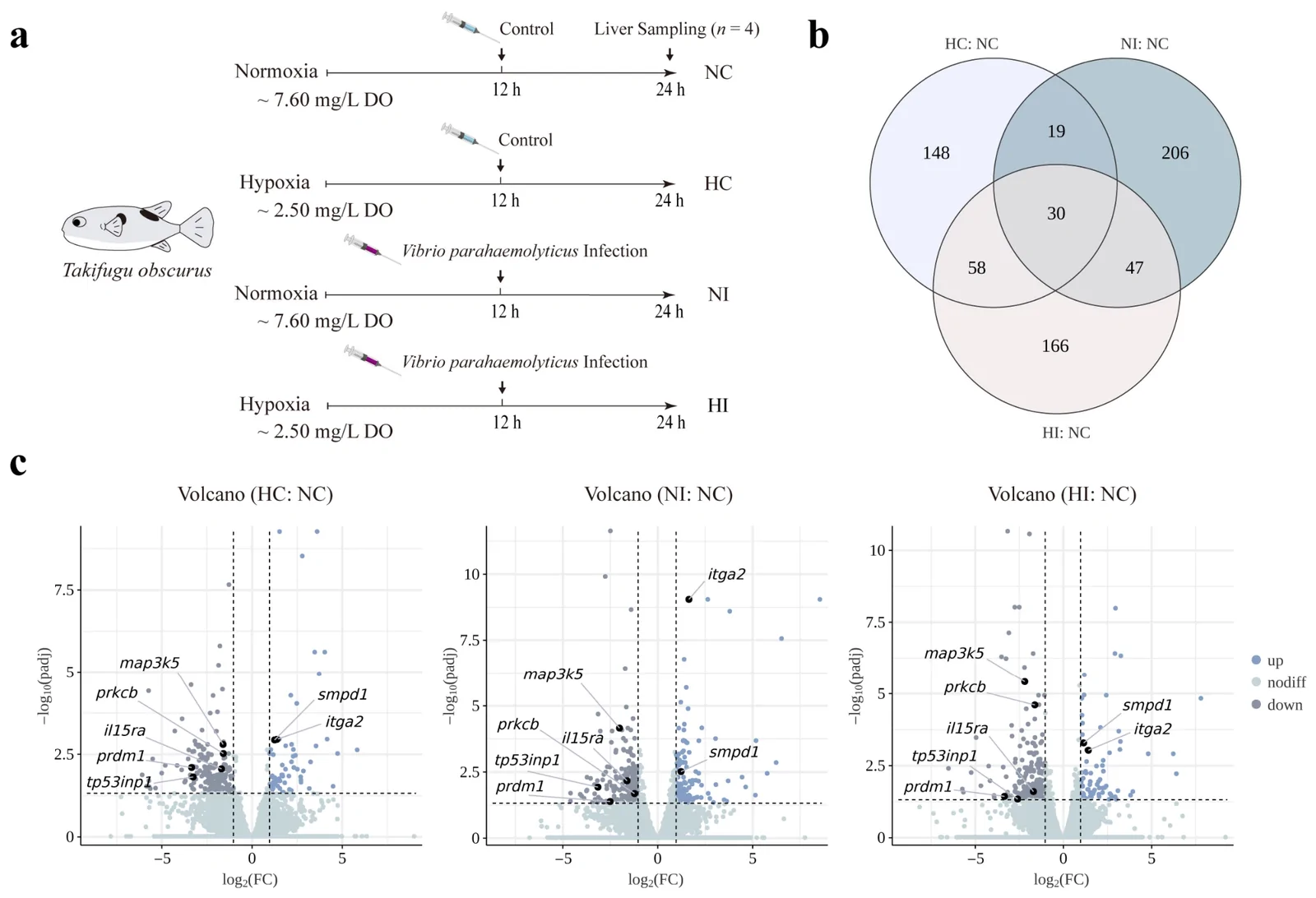
Hypoxia and *V. parahaemolyticus* infection trigger overlapping transcriptomic signatures in liver. (**a**) One hundred and sixty individuals of *T*. *obscurus* were randomly divided into four groups: normoxic control (NC), normoxic infected (NI), hypoxic control (HC), and hypoxic infected (HI). They were exposed to normoxia or hypoxia for 24 h, and injected with PBS or *V. parahaemolyticus* at 12 h. The livers were sampled for transcriptomic analyses at 24 h. (**b**) The Venn diagrams of DEGs of the three compared groups. (**c**) The volcano plots of DEGs of the three compared groups.

**Figure 2:**
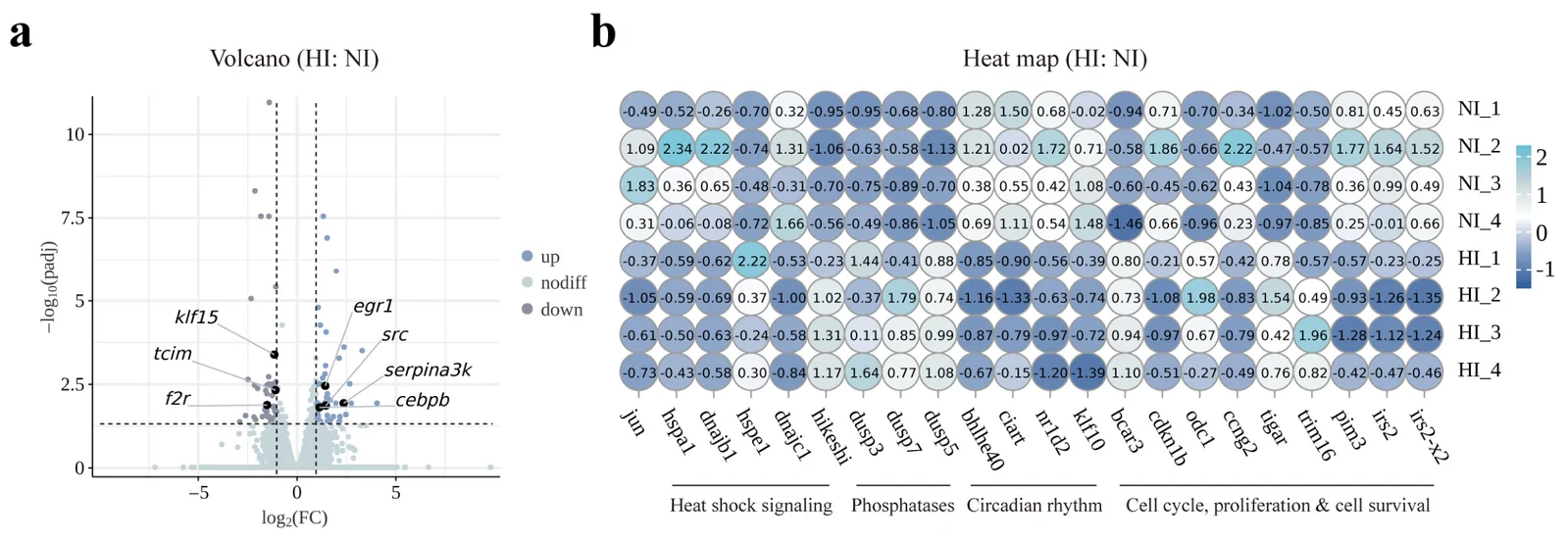
Transcriptomic analysis of hypoxia-induced immune dysregulation. (**a**) The volcano plot of DEGs associated with inflammatory cytokine regulation. (**b**) Clustering of DEGs showing four major functional modules: heat shock signaling, phosphatases, circadian rhythm, and cell cycle/proliferation/survival.

**Figure 3:**
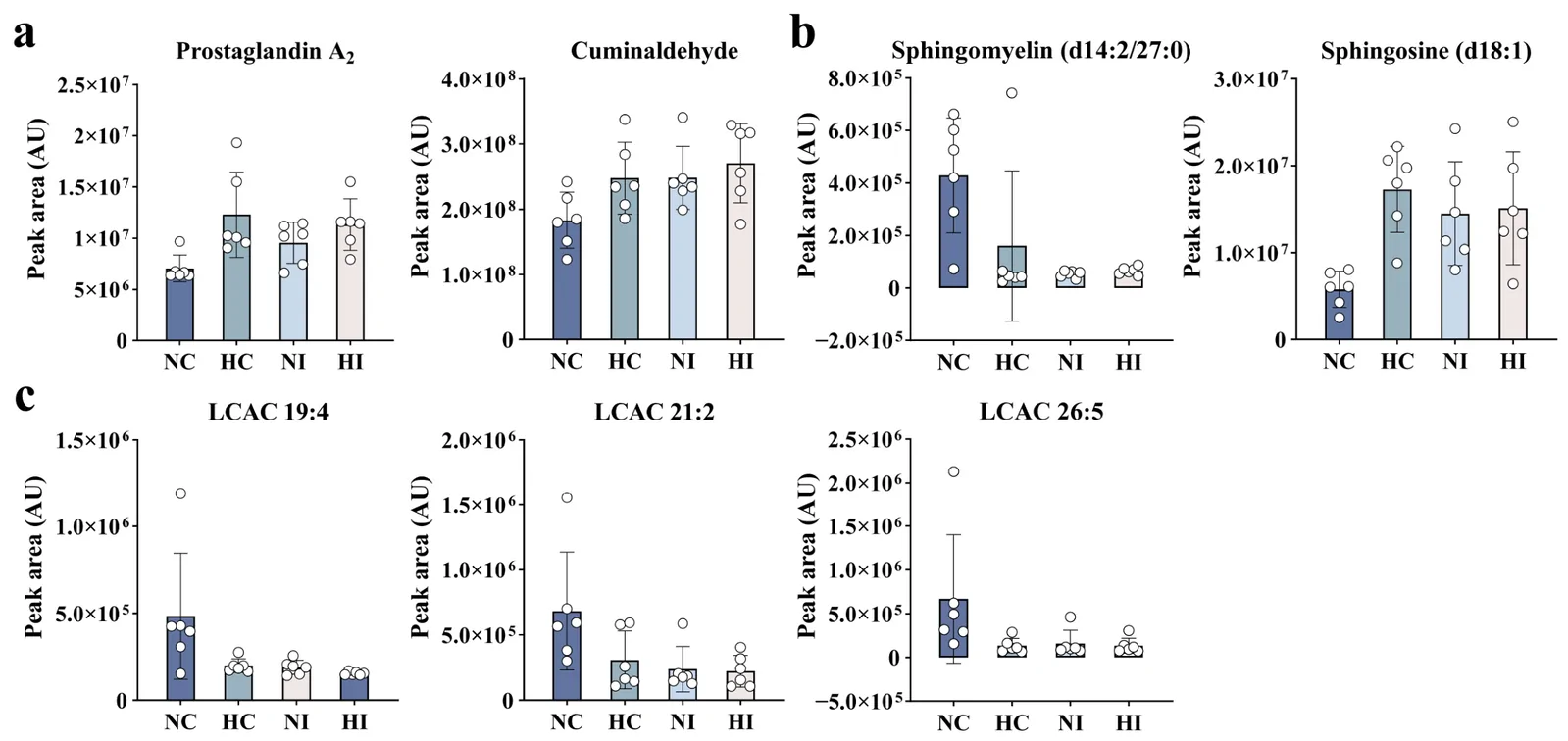
The overlapping metabolites were clustered into three categories (anti-inflammatory, sphingolipid metabolism and fatty-acid oxidation). (**a**) Prostaglandin A_2_ and cuminaldehyde might contribute to anti-inflammatory effects. (**b**) Sphingomyelin and sphingosine are related to sphingolipid metabolism. (**c**) The dynamic changes in LCACs may be associated with fatty-acid oxidation and anti-inflammatory signaling. AU: arbitrary unit. Data are presented as the mean ± SD (*n* = 6 per group). Statistical comparisons between each treatment group and the NC group were performed using Student’s *t*-test. All treatment groups exhibited significant differences relative to the NC group (*p* < 0.05). The exact *p*-values for each metabolite are provided in Supplementary Table S11.

**Figure 4:**
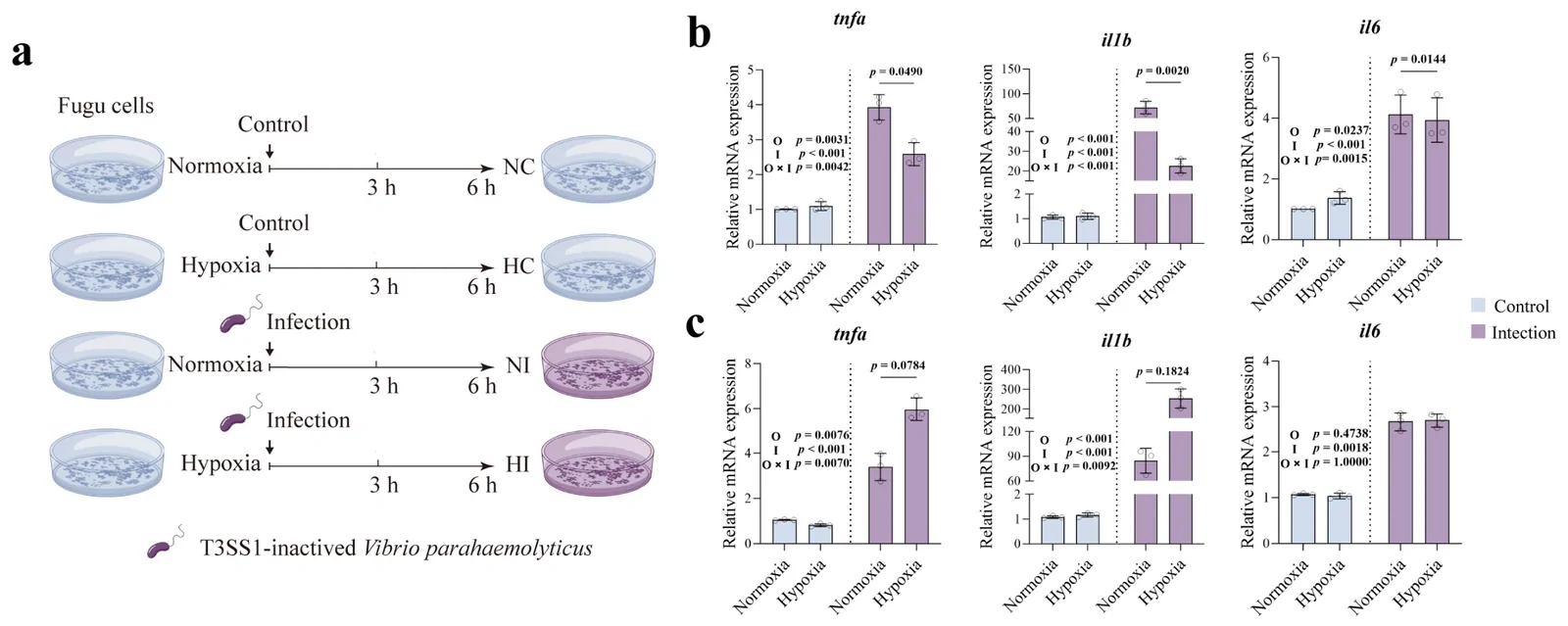
Oxygen tension is associated with altered mRNA expression of pro-inflammatory cytokines in Fugu cells induced by *V. parahaemolyticus*. (**a**) Experimental design: Fugu cells were infected with *V. parahaemolyticus* at an MOI of 10. Oxygen tension for normoxia and hypoxia was maintained at 21% O_2_ and 1% O_2_, respectively. (**b**) mRNA expression of *tnfa*, *il1b*, and *il6* was analyzed at 3 h post-infection. (**c**) mRNA expression of *tnfa*, *il1b*, and *il6* was analyzed at 6 h post-infection. Data are presented as the mean ± SD of three independent experiments. Aligned rank transformed two-way ANOVA with Tukey’s HSD post hoc test was applied to evaluate the main effects of oxygen tension (O), bacterial infection (I), and their interaction (O × I). The *p*-values for the main effects and interaction, as well as the pairwise comparison between NI and HI (where applicable), are shown in each panel.

**Table 1:** Primers for reference gene and pro-inflammatory cytokines.

Primers	Sequence 5′ to 3′	Amplicon Size (bp)	Amplification Efficiency (%)	Source
*rpl13*-F	GTAACAGGTCCACAGAATCCC	117	94	[22]
*rpl13*-R	CCTCAGTGCTGTCTCCCTTC			
*tnfa*-F	TACAGATCCCCACTACACGC	80	90	This study
*tnfa*-R	CTTCATCGAAGCTGCCTTCTAA			
*il1b*-F	GACACCATCGTGGAAGAGCAA	186	97	This study
*il1b*-R	CACTTTGCGACATTCCGAGC			
*il6*-F	AGCTGGAAAACAAGGTGAGGGA	158	91	This study
*il6*-R	GCAGTCGACCAGGAAGTAGTG			

## Data Availability

The transcriptomic and metabolomic data have been deposited in the China National GeneBank Database (CNGB, https://db.cngb.org/) under accession numbers CNX0467832 and CNP0002259, respectively. For further inquiries, please contact the corresponding author.

## Supplementary Materials

The following supporting information can be downloaded at: https://www.mdpi.com/article/10.3390/ani16203174/s1, Figure S1: *rpl13* remained stably expressed as a reference gene under hypoxia, *V. parahaemolyticus* infection, and combined hypoxia and infection stressors; Figure S2: *V. parahaemolyticus* exhibits strong cytotoxicity and rapidly kills Fugu cells; Table S1: Summary of alignment statistics; Table S2: HC vs NC DEGs; Table S3: NI vs NC DEGs; Table S4: HI vs NC DEGs; Table S5: HI vs NI DEGs; Table S6: DESeq2_interaction_results; Table S7: DESeq2_NI_vs_NC_normoxia_infection; Table S8: DESeq2_HI_vs_HC_hypoxia_infection; Table S9: DESeq2_HC_vs_NC_control_hypoxia; Table S10: DESeq2_HI_vs_NI_infected_hypoxia; Table S11: Statistical summary and exact *p*-values of the inflammation-associated metabolites shown in Figure 3.
